# A Unique Endoscopic Presentation of Colon Metastases From Primary Invasive Lobular Carcinoma of the Breast

**DOI:** 10.7759/cureus.37896

**Published:** 2023-04-20

**Authors:** Brendan R Martino, Victoria Mank, Salvatore Mignano, Zachary Neubert

**Affiliations:** 1 Department of Internal Medicine, Tripler Army Medical Center, Honolulu, USA; 2 Department of Pathology, Tripler Army Medical Center, Honolulu, USA; 3 Department of Gastroenterology, Tripler Army Medical Center, Honolulu, USA

**Keywords:** endoscopy, diaphragmatic stricture, colon metastasis, breast cancer, invasive lobular carcinoma

## Abstract

Breast cancer is the leading cause of female malignancy-associated death worldwide. The most common sites of metastases are the lung, liver, brain, and skeleton. A 68-year-old female with invasive lobular carcinoma metastatic to the axial skeleton was found to have new skin and colonic metastases discovered on serial surveillance positron emission tomography-computed tomography scans. The colonic metastases did not present with any gastrointestinal symptoms and did not form exophytic masses, which are typically associated. Instead, her colonic metastases presented as unusual diaphragm-like strictures within the left colon discovered on endoscopy, which is a relatively rare phenomenon. This case raises awareness of and elucidates new manners of presentation of metastatic invasive lobular carcinoma within the colon.

## Introduction

Breast cancer is the most common malignancy in women and the second leading cause of cancer-related deaths in the United States [1]. Treatment usually requires multi-specialty involvement employing a multimodal approach to therapy, including medical, radiologic, and surgical interventions. Despite these interventions, recurrence and metastasis can occur. Breast cancer is most known for metastasizing to the skeleton, lungs, liver, and brain [2]. Colonic involvement is a rare occurrence. Of patients with colonic involvement, invasive lobular carcinoma (ILC) is the most common form implicated, with associated gastrointestinal symptoms, and is usually found on colonoscopy as an obstructive mass [3,4]. This case demonstrates an asymptomatic patient with the presence of colonic breast cancer metastasis resulting in multiple diaphragmatic distal colon strictures without the typical exophytic character. There have been previously documented cases in the literature of similar colonic stricturing caused by ILC metastases [5]. In these cases, however, the patients all presented with various gastrointestinal symptoms, which is a distinguishing element of our case.

## Case presentation

A 68-year-old female with no significant medical history presented for evaluation of a palpable right breast mass in 2014. The painless mass was discovered three weeks prior on self-exam and was without any nipple discharge, retraction, or skin changes. Mammogram revealed a 2.3 cm right breast mass with a Breast Imaging-Reporting and Data System (BI-RADS) score of 5, indicating high suspicion for malignancy. A single enlarged 1.5 cm right-sided lymph node was also appreciated on imaging. A mammogram of the left breast revealed a 3 mm mass with a BI-RADS score of 4, indicating suspicion of malignancy. Ultrasound (US)-guided core biopsy of the right breast mass and the enlarged sentinel lymph revealed that the mass was estrogen receptor-positive (ER+), progesterone receptor negative (PR-), and equivocal 2+ amplified human epidermal growth factor receptor 2 (HER2) ILC. The ILC demonstrated diffuse infiltration into the surrounding adipose tissue and invasion into the capsule of the sentinel lymph node qualifying for the American Joint Committee on Cancer (AJCC) stage IIB (T2-N1-M0). She also underwent a US-guided core needle biopsy of her left breast mass. Core biopsy showed AJCC stage I (T1aN0i-M0) invasive ductal carcinoma (IDC) that was ER+, PR-, and HER2-. Given these findings, it was decided to proceed with bilateral mastectomy with right axillary lymph node dissection, left sentinel lymph node biopsy, and immediate reconstruction. The right breast confirmed ILC with negative surgical margins and no malignancy in the 15 axillary lymph nodes dissected. The left breast confirmed IDC with negative surgical margins, and the sentinel lymph node was negative for malignancy. She was followed by medical oncology and started on anastrozole in March 2015, eventually transitioning to exemestane in January 2017 due to myalgias.

**Figure 1:**
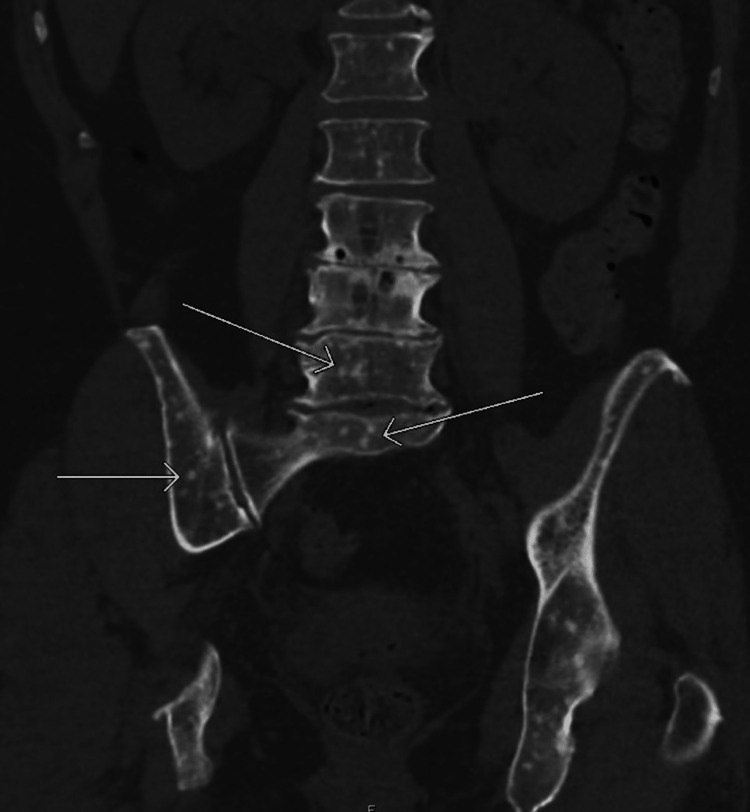
Coronal view of CT renal scan without signs of urolithiasis. Evidence of diffuse osseous lesions suspicious for metastasis throughout the axial skeleton (white arrows).

**Figure 2:**
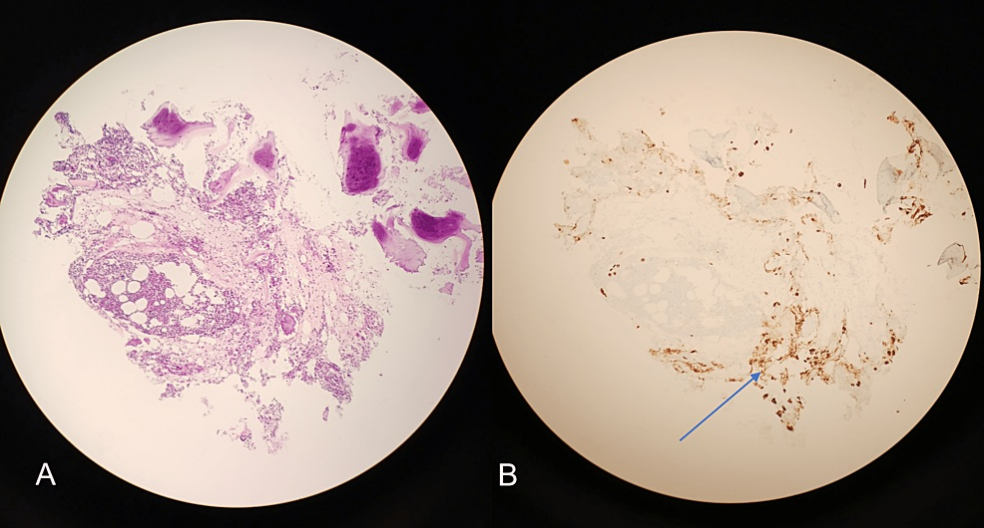
CT-guided core bone biopsy. (A) Hematoxylin & eosin staining of the biopsy specimen. (B) Immunohistochemistry of the same specimen with cytokeratin staining showing epithelial tumor cells of invasive lobular carcinoma within the bone marrow (blue arrow).

In August 2019, she developed flank pain, with computed tomography (CT) renal imaging demonstrating diffuse sclerotic lesions in the axial skeleton, raising concern for osseous metastasis (Figure 1). Baseline cancer antigen 15-3 (CA 15-3) and cancer antigen 27-29 (CA 27-29) were elevated at 147.9 U/ml (normal < 30 U/ml) and 114.0 U/ml (normal < 38 U/ml), respectively. A positron emission tomography/computed tomography (PET/CT) and CT-guided bone lesion biopsy confirmed ILC metastasis on immunohistochemistry with cytokeratin and showed ER+, PR-, and 3+ amplified HER2 expression in November 2019 (Figure 2). She began paclitaxel, trastuzumab, pertuzumab, and fulvestrant in January 2020. By July 2020, she developed skin metastases with worsening serum CA 15-3 and CA 27-29 tumor markers to 198 U/ml and 184 U/ml, respectively. However, there was no progression of disease on PET-CT that same month, and it was decided to continue her current chemo regimen.

**Figure 3:**
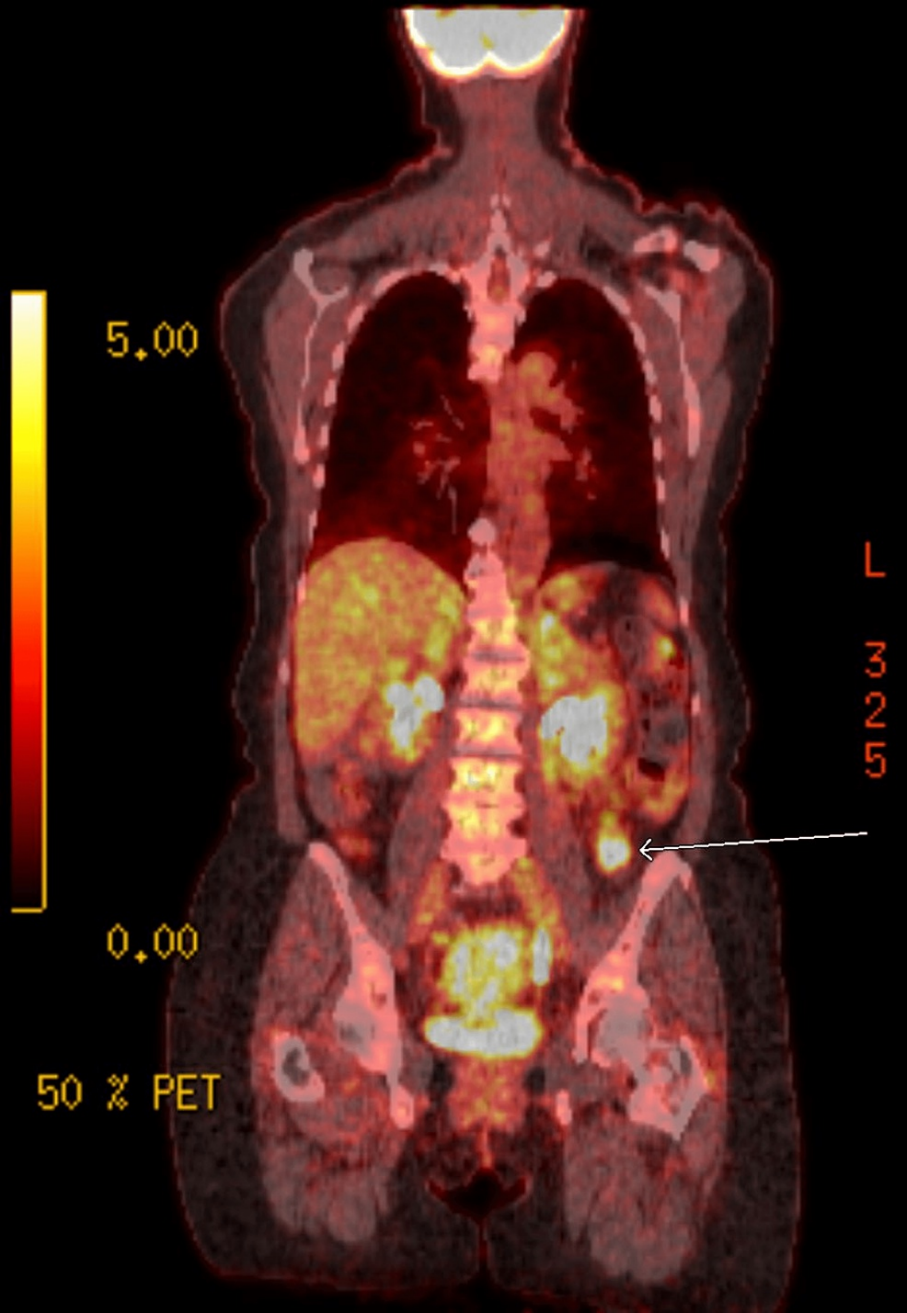
Coronal view positron emission tomography/computed tomography of the abdomen showing hypermetabolic activity in the distal colon (white arrow).

**Figure 4:**
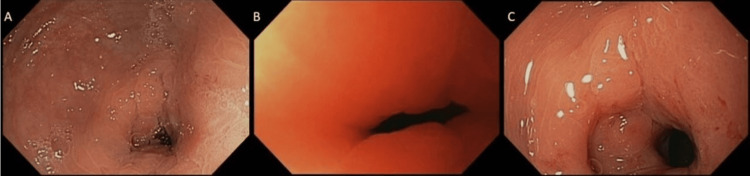
(A) Endoscopic view of the 20 cm colonic diaphragmatic stricture showing normal overlying mucosa without erythema or exophytic mass. (B) Endoscopic view of the 25 cm colonic stricture. (C) Endoscopic view of the 40 cm colonic stricture after the biopsy was taken at the 3 o’clock position.

**Figure 5:**
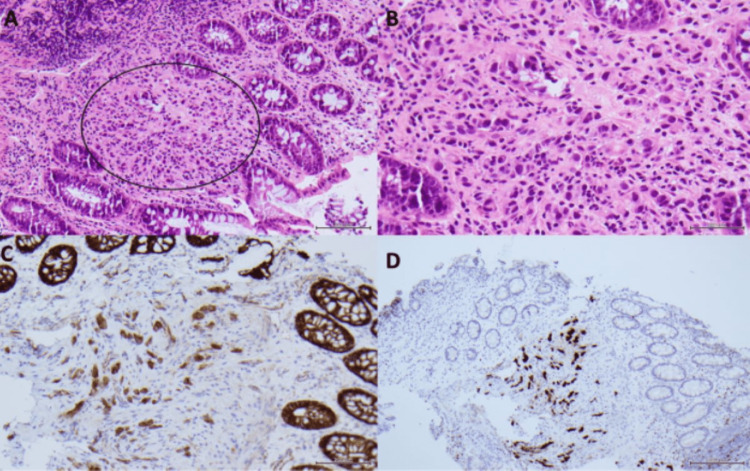
Twenty-centimeter colon biopsy pathology. (A) Single cells of invasive lobular carcinoma (ILC) (black circle) infiltrating between intestinal glands. (B) Mild inflammatory response with carcinoma cells that are plasmacytoid and have large nuclei. (C) In contrast to the normal membranous pattern, the ILC cells show granular, cytoplasmic staining with p120. This cytoplasmic pattern implies a loss of e-cadherin, an adhesion protein that anchors p120 to the membrane. Loss of e-cadherin expression and gain of purely cytoplasmic p120 expression are characteristics of ILC. (D) GATA3, a breast lineage marker, shows strong nuclear positivity in ILC. GATA3 is negative in the surrounding intestinal epithelium.

A PET/CT in October 2020 showed little change in her bony metastases, with new hypermetabolic activity in the colon and bowel wall thickening (Figure 3). She denied constipation, diarrhea, abdominal pain, nausea, melena, hematochezia, hematemesis, and tenesmus, and averaged one to two bowel movements per day. The previous colonoscopy in 2012 was unremarkable, with a repeat in December 2020 revealing a diaphragm-like stricture at 20 cm of scope insertion (Figure 4A). A neonatal gastroscope was then required to traverse two other diaphragm-like strictures within the sigmoid and descending colon, all with normal intervening colonic mucosa (Figures 4B, 4C). There were no exophytic masses found during the endoscopic evaluation of the left colon. Biopsies were obtained from each of the three colonic strictures. Biopsies from the colonic stricture at 20 cm showed infiltrative cells in the submucosa that were highlighted with GATA-3 and p120 immunohistochemical stains, an immunophenotype consistent with metastatic lobular breast carcinoma (Figure 5). Additionally, a chronic, granulomatous inflammatory response was seen in the surrounding colonic mucosa. The 25 cm stricture biopsies showed the presence of granulomas and colitis with the 40 cm stricture biopsies showing only colitis. She was referred to the colorectal surgeon for diverting ostomy formation, but due to a lack of obstructive symptoms, surgery was deferred and the surgeon opted for prophylactic medical management of constipation.

**Figure 6:**
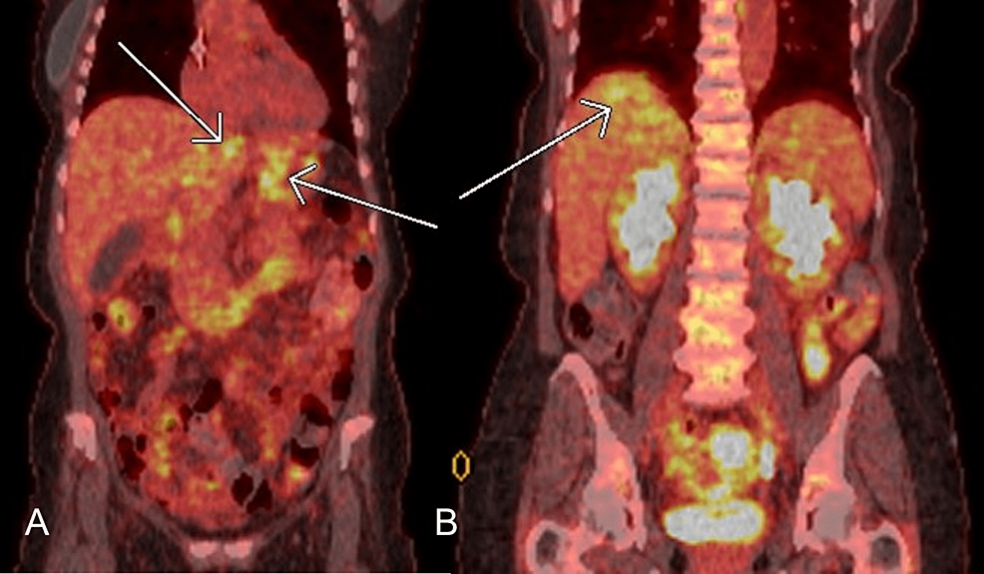
(A) Coronal view from positron emission tomography-computed tomography (PET-CT) showing new hypermetabolic activity in the left lobe of the liver (left white arrow) and gastric body (right white arrow). (B) Coronal view from PET-CT showing new hypermetabolic activity in the right lobe of the liver (white arrow).

**Figure 7:**
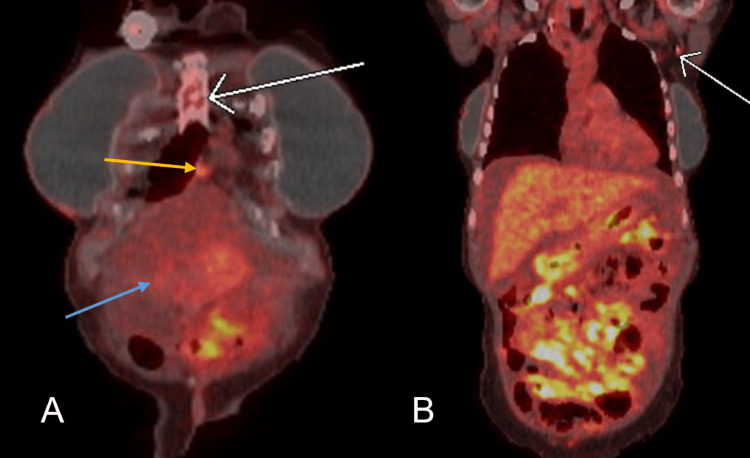
(A) Coronal view from positron emission tomography-computed tomography (PET-CT) showing worsening hypermetabolic retrosternal (white arrow) and pericardial (yellow arrow) lymph nodes. Also showing an interval increase in mildly metabolic ascites (blue arrow). (B) Coronal view from PET-CT showing new hypermetabolic activity in the left axillary lymph nodes (white arrow).

She had a repeat PET/CT in January 2021, which demonstrated persistent hypermetabolic activity in the colon, and also found new hypermetabolic activity in the gastric wall and in both lobes of the liver, indicating the progression of the disease (Figure 6). Given her biopsy-proven disease progression, her treatment regimen was switched from her current trastuzumab/pertuzumab to ado-trastuzumab. Due to her skeletal metastases and her increased risk of pathologic fractures, she was started on denosumab in addition to fulvestrant. In October 2021, she had evidence on PET/CT of disease progression, and she was switched to fam-trastuzumab. She was continued on this regimen for 15 cycles until a repeat PET/CT in November 2022 showed new disease progression with a new rise in CA 15-3 and CA 27-29 tumor markers to 430 U/l and 621 U/ml, respectively (Figure 7). She was switched to palbociclib, and a Guardant 360 liquid biopsy was sent, which showed the presence of BRAF gene mutation. She continued to have clinical deterioration and was deemed to have a life expectancy of less than six months. Palliative care was engaged, and it was decided to transition to hospice.

## Discussion

There are many possible etiologies for stricturing within the colon. Classically, it is due to radiation exposure or inflammatory bowel disease, neither of which were present in this patient [6,7]. There have also been reported cases of non-steroidal anti-inflammatory drugs (NSAID)-induced enterocolitis and stricturing within the colon [8]. Malignancy was also considered one of the possible causes of her endoscopic findings. Primary colonic malignancy versus metastatic spread from her known breast cancer were the two main differentials.

In general, breast cancer metastasis to the colon is rare. In cases that do, ILC is the most common involving 4.5% of all ILC cases vs. 0.2% of all IDC cases, and may appear as either inflammatory bowel disease or an obstructive or polypoid mass on endoscopy [9,10]. The presence of gastrointestinal involvement in breast cancer patients usually indicates systemic disease and poor prognosis with an average survival of six to 16 months [2,11,12]. Takeuchi et al. found that surgical intervention of these patients does not provide a mortality benefit without signs of obstruction or perforation [12].

Even though it is uncommon to see gastrointestinal involvement in breast cancer patients, it is extremely rare for a patient to present with colonic stricturing instead of an exophytic mass. The rarity of this phenomenon is likely due to the fact that the primary pathway of breast cancer metastasis to the colon is via hematogenous dissemination with 80% of the colonic blood flow going to the mucosal and submucosal layers [13]. Thus, resulting in the vast majority of metastatic ILC cells depositing within the mucosal and submucosal layers and growing as an exophytic mass. The remaining 20% supplies the muscularis layer [13]. This may provide an explanatory mechanism for our patient’s unusual presentation, as her malignancy may have deposited within the muscularis layer of the colon wall and grown circumferentially leading to progressively worsening colonic stricturing. This deeper deposition within the colonic wall poses further challenges, including allowing cancer to grow undetected for longer periods of time and also evade detection if only superficial biopsies are obtained on colonoscopy. There is also the possibility of lymphatic spread to the colonic and mesenteric lymph nodes from one of her other metastatic sites. There have been documented cases of breast cancer metastasis via lymphatic spreading [14]. However, given the directionality of lymphatic flow, it is less likely to be the cause of our patient’s colonic metastases. There have been reported cases of rectal, colonic, and small bowel stricturing secondary to ILC [5,15,16]. Nevertheless, our case is unique in that our patient presented without any gastrointestinal symptoms and had multiple diaphragm-like strictures with normal overlying mucosa found on colonoscopy.

Given the similar appearance of the strictures in our patient, it is likely that they were all caused by breast metastases versus multiple disease processes. The difference in histologic findings between the three colon biopsies likely indicates varying ages of the metastases versus sampling discrepancy of the biopsy specimens. The colitis that was present in all three of the biopsy specimens was likely induced by her current chemotherapy regimen at the time of colonoscopy rather than her metastatic malignancy. Gastrointestinal injury and diarrhea are common and well-documented side effects of pertuzumab therapy believed to be due to drug-induced mucositis [17]. Up to 70% of patients receiving pertuzumab therapy develop gastrointestinal toxicity, particularly when used with other chemotherapy agents [17]. This was established in the CLEOPATRA trial, which showed improved mortality in patients with locally recurrent, unresectable, or metastatic HER2+ breast cancer when treated with pertuzumab in addition to trastuzumab and docetaxel; however, 68% of these patients developed gastrointestinal toxicity and diarrhea [18].

## Conclusions

Breast cancer is the most common female malignancy in the United States and the leading cause of female cancer death in the world. While breast cancer metastasis to the colon is rare, it carries a very rapid mortality. Not only can breast cancer appear as exophytic masses within the colonic lumen, but it can also present as diaphragmatic stricturing likely due to deposition within the muscularis layer of the colon wall. This can then result in poor early detection due to a lack of gastrointestinal symptoms. Furthermore, the depth from the luminal surface can make pathologic diagnosis from endoscopic biopsies difficult. While it is not possible to recommend increased interval screening colonoscopy on ILC patients without gastrointestinal symptoms, physicians should be cognizant of gastrointestinal metastatic risk in patients with ILC and have a low threshold to obtain imaging and colonoscopy if such symptoms arise.
